# Isolation and enrichment of PC-3 prostate cancer stem-like cells using MACS and serum-free medium

**DOI:** 10.3892/ol.2012.1090

**Published:** 2012-12-21

**Authors:** XIA SHENG, ZENG LI, DE-LIN WANG, WEN-BIN LI, ZHAO LUO, KE-HONG CHEN, JIAN-JIA CAO, CHAO YU, WU-JIANG LIU

**Affiliations:** 1 Department of Urology, The First Affiliated Hospital of Chongqing Medical University; Chongqing 400016;; 2 Life Science Research Institute of Chongqing Medical University, Chongqing 400016;; 3 Institute of Urology, Peking University First Hospital Street 8 of Xishiku, Xicheng, Beijing 100034, P.R. China

**Keywords:** prostate cancer, stem cell, PC-3 cell, CD133, CD44, magnetic bead cell sorting, serum-free medium

## Abstract

Prostate cancer stem-like cells (PCSLCs) are considered to be the ‘seed’ of prostate cancer. The aim of this study was to confirm that the PC-3 cells, which we isolated and enriched from PC-3 cells through magnetic bead cell sorting (MACS) and serum-free medium (SFM) culture, were PCSLCs. Combinations of MACS, flow cytometry (FCM), SFM and immunocytochemistry (ICC) were used to ensure the positive expression of CD133 and CD44 on PC-3 and sphere-forming cell membranes. Self-renewal, multi-potential differentiation, unlimited proliferation and permanency assays were also applied to indentify whether the PC-3 cells exhibited the characteristics of cancer stem cells (CSCs). As a result, there was a low proportion of PCSLCs in the PC-3 cells. In the FCM assay, the proportion of cells expressing CD133 or CD44 in the PC-3 cells was 0.51 and 0.31%, respectively. In addition, we found that the proportion of PC-3 cells sorted by MACS that expressed CD133 was significantly increased compared with that of the sphere-forming cells cultured in SFM (99.09 vs. 84.80%, P<0.05), while no difference was observed in the proportion of cells expressing CD44 between them (99.88 vs. 99.82%, P>0.05). The expression of PAP and AR as detected by western blot analysis of induced PCSLCs was significantly increased compared with that of uninduced PCSLCs (P<0.05); the proliferation capacity of PCSLCs was significantly higher than that of both the PC-3 cells (P<0.05) and induced PCSLCs (P<0.05). Furthermore, the PCSLCs that were isolated from SFM and MACS both demonstrated certain characteristics of stem cells and should be considered as stem cell-like. These data may hold potential for further exploring the role of PCSLCs.

## Introduction

Tumor cells may be divided into well-differentiated mature or immature precursor cells according to different sources. The latter are considered to be cancer stem cells (CSCs) (1,2). They possess unlimited proliferative multi-potential differentiation, renewal, potential tumorigenicity capacity and permanency (3,4); however, the proportion of CSCs in adult tumor cells is small. In 1997, Bonnet and Dick confirmed for the first time that the small proportion of human acute myeloid leukemic (AML) cells that expressed CD34 on their membranes were AML stem cells (5). This finding provided initial insights into the domain of stem cells. Subsequently, CSCs in breast (6), prostate (4), liver (7) and colon (8) cancer cells were isolated through their respective surface markers.

Based on the fact that the proportion of CSCs in adult tumor cells is small, Collins *et al* purified the prostate cancer stem cells (PCSCs) from prostate cancer by flow cytometry (FCM). The author demonstrated that ∼0.1% of prostate cancer cells possessed the CD44^+^/α_2_β1hi/CD133^+^ phenotype, which was independent of prostate cancer grading and staging (4). Numerous scholars have indicated that prostate cancer cells with the CD133^+^/CD44^+^ phenotype demonstrate certain stem cell characteristics, including renewal, multi-potential differentiation, unlimited proliferative capacity and permanency (9–11). However, little is known regarding whether cells with the CD133^+^/CD44^+^ phenotype are prostate cancer stem-like cells (PCSLCs). Therefore, in this study, we aimed to isolate and enrich PC-3 PCSLC cells by magnetic bead cell sorting (MACS) and serum-free medium (SFM) based on CD133 and CD44.

## Materials and methods

### Cell culture

The PC-3 cell line (derived from grade IV bone metastases of prostate adenocarcinoma in a white, 62-year-old male) was purchased from the Shanghai Cell Bank of the Chinese Academy of Sciences. The cells were cultured with Dulbecco’s modified Eagle’s medium (DMEM)/F12 (1:1) (HyClone Laboratories, Inc.; South Logan, UT, USA) and 10% fetal calf serum (Gibco-BRL; Carlsbad, CA, USA), in a 25-ml culture flask in an incubator at 5% CO_2_ and 37°C.

### SFM culture of spheres

PC-3 cell spheres were cultured with DMEM/F12 (1:1) supplemented with 20 *μ*g/l epidermal growth factor (EGF), 20 *μ*g/l basic fibroblast growth factor (bFGF) and 20 *μ*g/l leukaemia inhibitory factor (LIF) (Peprotech, Inc.; Rocky Hill, NJ, USA) in an incubator as previously described (11). The cells were observed each day using an inverted microscope. Medium (1 ml) was added on alternate days and half the medium was replaced biweekly. The spheres were suspended in the medium within 7–9 days; the suspension was drained into another flask and the culturing process was continued in the same way.

### MACS

The CD133^+^/CD44^+^ PCSLCs were obtained using the MACS kit according to the manufacturer’s instructions (Miltenyi Biotech, Bergisch Gladbach, Germany) (12). Briefly, total populations of adherent cells were enzymatically dissociated into a single cell suspension and counted to confirm the quantity of the whole cells. The cells were incubated with 100 *μ*l microbeads directly conjugated to mouse monoclonal anti-human CD133 antibody at 4°C for 30 min. Subsequently, the suspended cells were added to a MACS column that was placed in the magnetic field of a MACS separator (Miltenyi Biotech). The labeled CD133^+^ cells were retained on the column and the unlabeled cells were eluted; when the column was removed from the magnetic field, the magnetically retained CD133^+^ cells were collected as positively selected cells for further research (13). The CD44^+^ cells were obtained using the same method.

### FCM

FCM was performed at the FCM Laboratory, Chongqing Medical University. The collected PC-3 cells were named group 1; the collected spheres, which were cultured in SFM, were designated group 2 and the cells sorted by MACS formed group 3. Each group was incubated with 20 *μ*l mouse anti-human monoclonal antibody of CD133-phycoerythrin (PE; eBioscience, San Diego, CA, USA) for 30 min at room temperature in the dark. This was followed by centrifugation for 5 min at 800 rpm, extraction with 300 *μ*l phosphate-buffered saline (PBS) and detection on the machine. This procedure was repeated three times for each group. The same method was also used for the antibody of CD44-phycoerythrin. Subsequent FCM analysis demonstrated <5% contamination by relevant antigen-expressing cells.

### Immunocytochemistry (ICC)

The expression of CD133 and CD44 was analyzed by immunofluorescence techniques. The isolated cell suspension was subjected to fixation for 20 min with 4% paraformaldehyde and sealed with goat serum to avoid non-specific binding. This was then incubated with the primary antibody overnight at 4°C, prior to incubation with combination fluorochrome-conjugated secondary antibody for 40 min at 37°C on the following day. Subsequently, staining with 4′,6-diamidino-2-phenylindole (DAPI) was performed for identification of the nuclei, and slides were sealed with 50% glycerin.

### Proliferation assay

Collected spheres, PC-3 cells, cells with a CD133^−^/CD44^−^ phenotype and differentiated spheres comprised groups 1, 2, 3 and 4, respectively. Each group was seeded at a low density (2×10^3^ cells/200 *μ*l), the zero setting was put in place by a blank medium, and then cells were incubated in an incubator for 7 days. The samples were randomly removed each day and the cell number was determined by a microplate reader. A proliferation curve was then generated accordingly and the doubling time of cells was calculated using the Patterson formula: T_d_=T×lg2/lg(N_t_/N_0_); where T_d_ indicated doubling time, T indicated days, N_t_ indicated the number of cells on the last day, N_0_ indicated the numbers of cells on the first day.

### Differentiation assay

The PCSLCs were cultured with DMEM/F12 (1:1) supplemented with 5 *μ*g/l TGF-β (Peprotech, Inc.) and 1% fetal calf serum (14). The PCSLCs that were cultured without TGF-β were used as a control group. The PCSLCs were incubated for 4 days and their morphology was observed by an inverted microscope.

### Western blot analysis

Cell lysates were prepared by a mixture of phenylmethylsulfonyl fluoride (PMSF) and protease inhibitor (1:99). Proteins were separated by gel electrophoresis and then transferred to a 0.45-*μ*m polyvinylidene fluoride (PVDF) membrane. The primary antibody (dilution, 1:200) and horse-radish peroxidase (HRP)-labeled secondary antibody (dilution, 1:5,000) were added. The membrane was placed in the Vilber Lourmat (Bio-Rad; Hercules, CA, USA) for enhanced chemiluminescence (ECL) development. The density values were determined by: Target protein/β-actin.

### Statistical analysis

Data were presented as mean ± standard deviation. A Student’s two-tailed t-test was used to compare two groups, while a one-way analysis of variance (ANOVA) was used to compare multiple groups. P<0.05 was considered to indicate a statistically significant difference.

## Results

### Expression of CD133 and CD44 in PC-3 cells before and after MACS sorting by FCM

**Figure 1:**
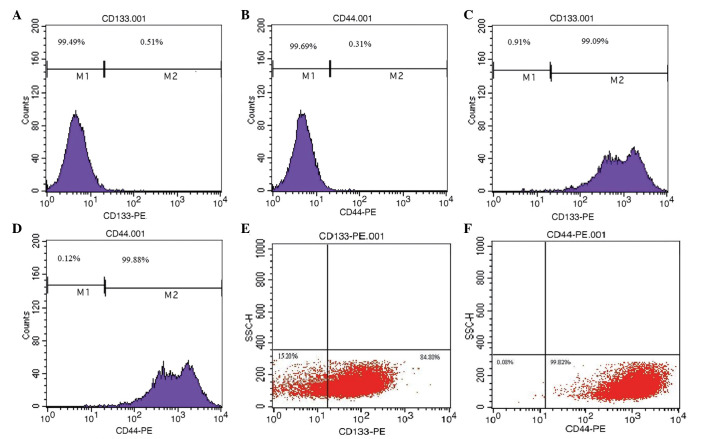
Surface marker expression analysis by flow cytometry (FCM). Proportion of PC-3 cell membranes expressing CD133 (A); CD44 (B); CD133, following magnetic bead cell sorting (MACS) (C) and CD44, following MACS (D). Proportion of sphere-forming cell membranes expressing CD133 (E) and CD44 (F).

We investigated whether there was specific CD133 or CD44 expression on the PC-3 cell membranes, and found that the proportion of cells expressing CD133 and CD44 was 0.51 and 0.31%, respectively, by FCM (Fig. 1A and B). Therefore, the proportion of cells expressing CD133 and CD44 was in accordance with a small group of cells, based on these results.

To confirm the existence of the CD133^+^/CD44^+^ phenotype in PC-3 cells, we purified the cells by MACS and demonstrated that the proportion of cells expressing CD133 and CD44 was 99.09 and 99.88%, respectively (Fig. 1C and D). The proportion of cells with the CD133^+^/CD44^+^ phenotype was 0.53±0.07 (Table I).

### Formation of PCSLC spheres cultured in SFM

**Figure 2:**
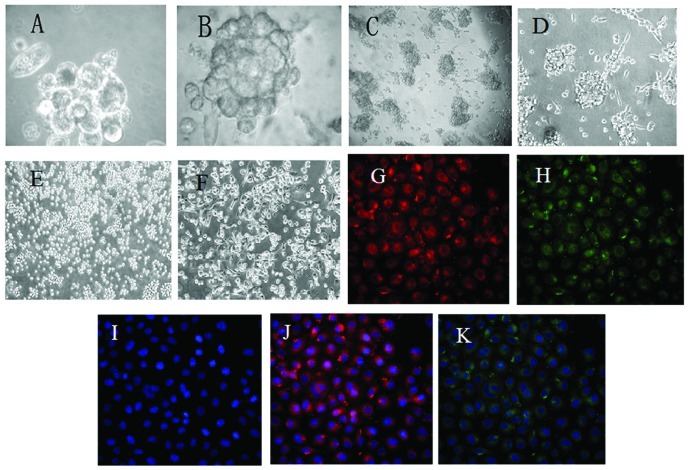
Formation process of prostate cancer stem-like spheres (PCSLSs) in serum-free medium (SFM). Three-day (A) and seven-day (B) phases of spheres in SFM (×400). Four-day phase of spheres (C), and one-day (D), two-day (E) and four-day (F) phases of TGF-β-induced spheres (×100). A comparison of sphere-forming cell immunofluorescence staining between CD133 (G), CD44 (H) and the nuclei phase (I) (×200). The merge phase of CD133 and CD44 is demonstrated in (J) and (K), respectively.

PCSLC spheres were capable of forming in SFM. The majority of cells with the CD133^+^/CD44^+^ phenotype were growing cultured in SFM, while few cells exhibited apoptosis. After three days, 5–15 differently sized spheres with a capacity of refraction had formed and were loosley combined (Fig. 2A). Subsequently, the spheres enlarged and the connection between the cells became tighter. With the extension of culture time, new cells were born by budding on the spheres that became rounder within four days (Fig. 2B). The spheres passaged and formed new irregular spheres, which became regular after one week (Fig. 2C).

### Expression of CD133 and CD44 on sphere-forming cell membranes detected by FCM

To determine whether the spheres that we had cultured in SFM were PCSLCs, we detected the expression of CD133 and CD44 on sphere-forming cell membranes by FCM; the proportion of cells expressing CD133 and CD44 was 84.8 and 99.82%, respectively (Fig. 1E and F). The expression rate of CD133 on sphere-forming cell membranes was close to that in PC-3 cells isolated by MACS (15), but the difference between them was significant (P<0.05). However, the expression of CD44 on sphere-forming cell membranes was almost equivalent to that in PC-3 cells isolated by MACS, and the difference was not significant (P>0.05).

### ICC analyses of CD133 and CD44 expression on sphere-forming cell membranes

To further assess whether CD133 and CD44 were expressed on sphere-forming cell membranes, following several subcultures, a high expression level of CD133 and CD44 was observed by ICC (Fig. 2G and H), and the nuclei had stained blue (Fig. 2I). In the merge phase (Fig. 2J and K), CD133 and CD44 were mainly expressed on the membranes. On incubation with rhodamine (TRITC)-conjugated anti-rabbit IgG antibody, CD133 expressed a red fluorescence, while CD44 exhibited a green fluorescence when incubated with FITC-conjugated anti-rabbit IgG antibody. Thus, the CD133^+^/CD44^+^ subpopulation could be enriched and subcultured with permanent retention.

### Differentiation assays of prostate cancer stem-like spheres (PCSLSs)

**Figure 3:**
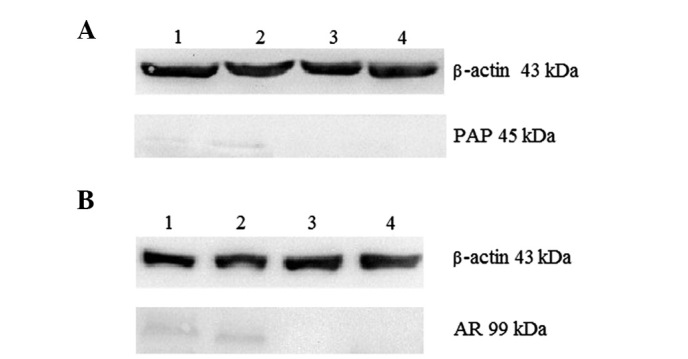
Western blot analysis of PAP (A) and AR (B). 1, spheres induced by TGF-β; 2, cells with the CD133^+^/CD44^+^ phenotype induced by TGF-β; 3, spheres and 4, cells with the CD133^+^/CD44^+^ phenotype.

The process of differentiation of spheres was evident following treatment with TGF-β. The morphological changes in the sphere-forming cells were marked in the one-, two- and four-day phases. The spheres remained suspended for the first 12 h, then after one day certain cells became adherent to the wall, with spindles and deformation evident (Fig. 2D). After two days, numerous cells were adherent and no marked differences in the morphology were observed, compared with the control group (Fig. 2E). All spheres had adhered to the wall after 4 days, with various forms evident (Fig. 2F). The results of the western blot analysis revealed that cells with the CD133^+^/CD44^+^ phenotype and spheres that had both been induced by TGF-β co-expressed PAP and AR (Fig. 3A and B); the values of PAP and AR expression were 0.192±0.011 and 0.473±0.064, respectively, while the control groups expressed neither of these proteins.

### PCSLSs exhibit a high proliferative capacity in vitro

**Figure 4:**
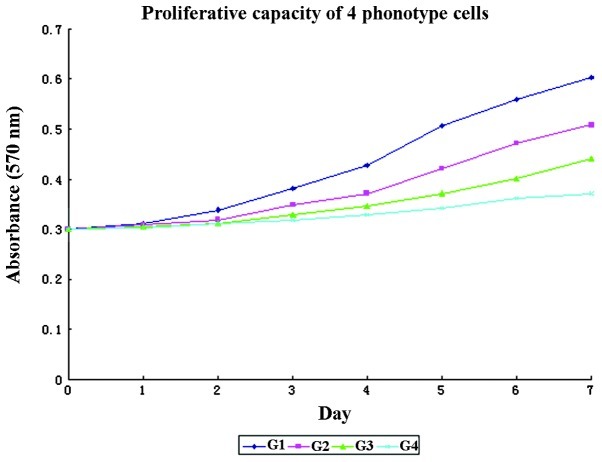
Proliferation assay of the four cell phenotypes: G1, sphere cells following several subcultures; G2, PC-3 cells; G3, cells with the CD133^+^/CD44^+^ phenotype following MACS; G4, sphere cells following induction by TGF-β.

To determine whether PCSLSs were different from PC-3 cells, we next observed the proliferative properties of four independent phenotypes *in vitro*. As demonstrated in Fig. 4, the categories comprised spheres, PC-3 cells, cells with a CD133^−^/CD44^−^ phenotype and differentiated spheres, as groups 1–4 (G1–4). G1 was the group with the highest proliferative capacity. A significant difference was observed between the absorbance levels of G1, 2 and 3 between the 1st and 7th days (P<0.05), while that of G4 was not signifcantly different, although the absorbance was higher on the 7th day (P>0.05). Significant differences were identified in the doubling times and cell numbers among G1–4; the doubling times were 23.24, 38.87, 57.79 and 133.01 h, while the cell numbers were 3×10^5^, 4×10^4^, 1.5×10^4^ and 4.8×10^3^ in G1–4, respectively (P<0.05).

## Discussion

CSCs are not only the origin of tumors but also the basis of tumor progression, resistance to therapy, metastasis and subsequent tumor recurrence (16,17). An increasing number of studies confirm that solid tumors and established cancer cell lines are organized in a hierarchy of heterogeneous cell populations, and that CSCs sustain the growth of cancer cells overall (18). Methods to identify and isolate CSCs include cell sorting based on surface markers and staining of cell subpopulations forming tumor cell spheres (19–21).

Although the majority of tumor cells may be eliminated with the current treatment methods, difficulty remains in treating androgen-independent prostate cancer (AIPC), which develops in the majority of prostate cancer patients. If the stem cell hypothesis is accepted, traditional treatment is not capable of preventing tumor recurrence, as only the differentiated mature cells and not a small population of cells may be killed (11). It has been suggested that prostate cancer is a stem cell disease (22) and that human PCSCs exhibit the same traits as CSCs, including renewal, multi-potential differentiation, unlimited proliferation capacity and permanency. Therefore, effective therapy for prostate cancer should target PCSCs. However, a wide gap in the knowledge remains, as fewer cells with the CD133^+^/CD44^+^ phenotype are available and there is a lack of powerful methods of identification. Herein, we established an effective scheme whereby PCSLCs may be isolated and enriched.

CD133 and CD44 are considered to be PCSC biomarkers. Guo *et al* demonstrated that Epcam+CD44^+^ prostate basal cells are able to form abundant spheres, while Epcam^+^CD44^−^ cells are not (23). Patrawala *et al* revealed that CD44^+^ prostate cancer cells possessed stem-like cell characteristics, including increased tumorigenicity, clonogenicity and metastatic potential (24). However, whether CD44 was the specific biomarker of CSC is unknown. Our results demonstrated that there was no significant difference in the expression of CD44 between PC-3 cells, following MACS, and sphere cells, while that of CD133 exhibited a significant difference. Therefore, we concluded that the hypothesis that CD133 is the specific biomarker of PCSCs remains controversial. Vander *et al* demonstrated that CD133^+^ prostate cancer cells exhibited stem cell properties, such as renewal and multi-potential differentiation, even after several subcultures (25).

Li *et al* found that prostate cancer cell holoclones contained self-renewing cells and could be continuously propagated (26). Holoclones have been demonstrated to exhibit a high renewing capacity under SFM culture, whilst retaining their holoclone morphology and demonstrating high tumorigenicity (27). These findings are concordant with our results, in which spheres that formed in SFM could be continuously propagated, whilst retaining their CSC-like characteristics. This shows that the method that we designed is able to isolate and enrich PCSLCs.

To further identify the PCSLCs that possessed the characteristics of CSCs, we conducted proliferation and differentiation assays. Certain studies have concluded that TGF-β regulates multiple cellular functions and influences normal prostate growth and differentiation (28,29). The untreated spheres exhibited the highest absorbance and the shortest doubling time; while spheres induced by TGF-β had the lowest absorbance, which may reflect that the proliferative rate of PCSLCs was higher than the proliferative rate of the cancer cells in a stage after differentiation. Following induction with TGF-β, the spheres exhibited various morphologies, while the PC-3 cells demonstrated no morphological changes. The PC-3 cell line has been demonstrated to possess characteristics of prostatic small cell carcinoma and to not express AR, while this was observed in the DU145 cells (30), and the mRNA level of PAP was almost undetectable in the PC-3 cells (31). In our study, the PAP and AR proteins were not expressed on PC-3 cells with the CD44^+^/CD133^+^ phenotype and sphere-forming cell membranes. However, following treatment with TGF-β, these cells co-expressed PAP and AR. Therefore, the PCSLCs may be induced into other differentiative cells, of which the subpopulations have not been confirmed in this study; thus, PCSCs may be considered to be the progenitor cells of these mature cells.

To conclude, PCSLCs may be considered to be PCSCs, which possess certain characteristics of all types of CSCs. The CD133+/CD44+ phenotype of PC-3 cells represented a small subpopulation of cells (∼0.53%) within the whole population of tumor cells. Additionally, the spheres were able to continue to proliferate and self-renew, while retaining their morphology. Furthermore, the PCSLCs exhibited multi-potential differentiation. Therefore, the processes of serum-free suspension culture and MACS may be used effectively to enrich and isolate PCSLCs, and to provide an inexhaustible source of genetically stable PCSCs for studies. Understanding how to eliminate cancer cells in the stationary phase that are considered to be PCSCs will lead to an effective treatment for prostate cancer, and bring benefit to AIPC patients.

## Figures and Tables

**Table I:** Proportion of the CD133^+^/CD44^+^ phenotype in PC-3 cells.

	Sorting times						
	1	2	3	4	5	6	7
PC-3 cell numbers before MACS (×10^7^)	3.5	4.0	2.1	4.4	4.0	7.0	6.0
CD133^+^/CD44^+^ cell number after MACS (×10^5^)	2.0	2.3	1.1	1.8	1.8	4.0	3.6
Proportion of CD133^+^/CD44^+^ (%)	0.57	0.58	0.52	0.41	0.45	0.57	0.60
